# Uveitis Associated with Monogenic Autoinflammatory Syndromes in Children

**DOI:** 10.1080/09273948.2023.2282610

**Published:** 2023-12-05

**Authors:** P. Maghsoudlou, A. R. Abraham, M. El-Ashry, C. Chew, N. Mohd, A. V. Ramanan, A. D. Dick

**Affiliations:** a Academic Unit of Ophthalmology, Translational Health Sciences, Bristol Medical School, University of Bristol, Bristol, UK; b Department of Paediatric Ophthalmology, Bristol Eye Hospital, Bristol, UK; c Department of Paediatric Rheumatology, University of Bristol, Bristol, UK; d School of Cellular and Molecular Medicine, University of Bristol, University Walk, Bristol, UK; e UCL Institute of Ophthalmology, London, UK; f NIHR - Biomedical Research Centre, Moorfields and UCL - Institute of Ophthalmology, London, UK

**Keywords:** Autoinflammation, cytokine, inflammasome, inflammation, uveitis

## Abstract

Monogenic autoinflammatory syndromes (MAISs), are caused by pathogenic genetic variants in the innate immune system, leading to dysregulation and aberrant inflammasome activation spontaneously or with minimal triggering. The diagnosis and treatment of MAISs can be intricate, relying on an increased recognition of potential differential diagnoses. This review examines the clinical features of MAIS, with a special focus on uveitis. It also evaluates treatment options and assesses the effects of activating molecular and cytokine pathways.

Monogenic autoinflammatory syndromes (MAISs) arise from disorders of the innate immune system. Familial Mediterranean fever (FMF), tumour necrosis factor (TNF) receptor-associated periodic fever syndrome (TRAPS), mevalonate kinase deficiency (MKD), and cryopyrin-associated periodic syndrome (CAPS) are the most extensively studied monogenic autoinflammatory conditions.^1^ The episodes of inflammation are caused by a dysregulated immune system, which leads to excessive production of pro-inflammatory cytokines, for example, interleukin-1β (IL-1β).

Inflammasomes are multiprotein complexes belonging to the family of pattern recognition receptors and are an integral part of the innate immune system.^2^ These multiprotein complexes can sense pathogen-associated molecular patterns (PAMPs) and damage-associated molecular patterns (DAMPs). A well-characterised PAMP is lipopolysaccharide (LPS), located on the outer cell wall of gram-negative bacteria. DAMPs originate from host cells, encompassing tumour or dying cells, as well as substances that cells release in response to different types of stress.

There are several inflammasomes, and in general, these complexes typically form around a cytoplasmic receptor belonging to the nucleotide-binding leucine-rich repeat-containing receptor (NLR) family (such as NLRP3; Figure 1), although other cytoplasmic receptors such as pyrin have been described. At present, a two-step framework is proposed to explain the commencement of NLRP3 inflammasome activation.^3^ The first step involves priming of the system via the NF-κB signalling pathway, which promotes transcription of NLRP3, and the inactive pro-IL-1β and pro-IL18. The second step involves activation of the inflammasome upon recognition of a microbial, danger, or homeostasis pattern by the receptor. NLRP3 connects with the adaptor protein ASC as a response and recruits procaspase-1, which leads to the self-cleaving activation of caspase-1 (Figure 1). The ensuing inflammatory response is highly dependent on caspase-1, which in turn results in cleavage of inactive pro-IL-1β and pro-IL-18 to the active IL-1β and IL-18. Caspase activation also leads to pyroptosis, a specific form of programmed cell death, that is executed through the cleavage of Gasdermin D. In Figure 1 illustrating the inflammasome activation, we highlight distinct locations where pathogenic variants lead to the development of MAISs. This figure highlights how particular genetic changes in the components of the inflammasome system can lead to the development of these autoinflammatory conditions.

**Figure 1.**
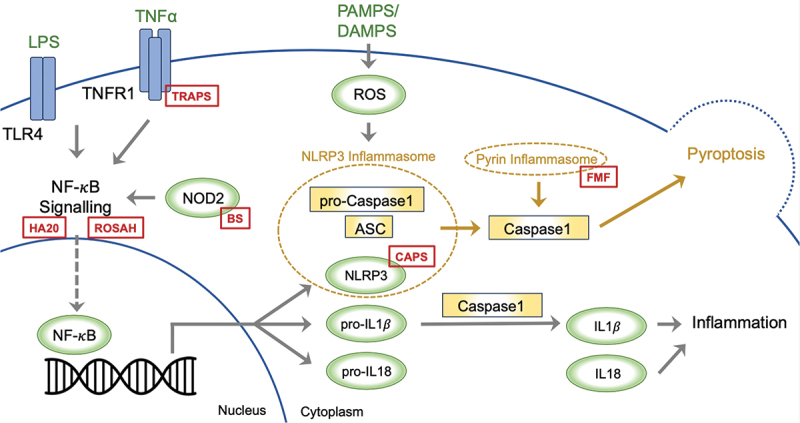
A two-step model has been proposed to explain the initiation of NLRP3 inflammasome activation. The first step entails system priming through the NF-κB signalling pathway, promoting the transcription of NLRP3, pro-IL-1β, and pro-IL-18. In the second step, the inflammasome activates upon the recognition of PAMPS or DAMPS. NLRP3 associates with the adaptor protein ASC, prompting the recruitment of procaspase-1, which leads to caspase-1 self-cleaving activation. The ensuing inflammatory response significantly hinges on caspase-1, facilitating the conversion of inactive pro-IL-1β and pro-IL-18 into active IL-1β and IL-18. Caspase activation also triggers pyroptosis; a specific form of programmed cell death. The red boxes depict the sites within inflammasome activation, where pathogenic variants drive the development of monogenic autoinflammatory syndromes; ASC: Apoptosis-Associated Speck-Like Protein, BS: Blau Syndrome, CAPS: Cryopyrin-Associated Periodic Syndromes, DAMPs: Damage-Associated Molecular Patterns, FMF: Familial Mediterranean Fever, HA20: Haploinsufficiency A20, IL: Interleukin, LPS: Lipopolysaccharide. NLRP3: nucleotide-binding leucine-rich repeat-containing receptor 3, NOD2: Nucleotide-Binding Oligomerisation Domain-Containing Protein 2, NF-κB: Nuclear Factor Kappa B, PAMPs: Pathogen-Associated Molecular Patterns, ROSAH: Retinal dystrophy, Optic nerve oedema, Splenomegaly, Anhidrosis, and Headache Syndrome, TLR4: Toll-Like Receptor 4, TNFα: Tumour Necrosis Factor Alpha, TNFR1: Tumour Necrosis Factor Receptor 1, TRAPS: Tumour Necrosis Factor Receptor-Associated Periodic Syndrome.

Variations in clinical presentation among patients with MAISs stem from both the specific pathogenic variant, as well as the distinctive cellular distribution of a given inflammasome and its associated substrates. The diagnosis of MAISs can be intricate, relying on an increased recognition of potential differentials. In this review, we delve into the clinical characteristics of MAISs, with a specific emphasis on uveitis, and explore the corresponding treatments for each condition. Our aim is to elucidate how these distinct molecular and cytokine pathways influence the selection of appropriate treatments for each condition.

## Familial Mediterranean fever (FMF)

FMF is an autosomal recessive condition caused by pathogenic variants of the MEFV gene, encoding the pyrin protein.^6^ FMF is predominantly seen in Mediterranean populations with highest prevalence in Arab, North African Jewish, Middle Eastern, Turkish, and Armenian ethnicities.^7^ In Armenian and Israeli populations, the carrier rate varies from 1 in 4 to 1 in 8. However, the disease also occurs in other populations and is not exclusive to places with higher prevalence, displaying variation in genotype and phenotype (Table 1).^8^

A pathogenic variant in the *MEFV* gene was the first to be implicated within the pathophysiology of FMF or any other autoinflammatory disease and is also relevant in Behçet’s disease and ankylosing spondylitis.^9^ *P*yrin is an innate immune system protein found predominantly in myeloid lineage cells, fibroblasts, and dendritic cells. Pyrin’s role as a pattern recognition receptor allows it to detect pathogen virulence activity and stimulate a pyrin inflammasome, which leads to an inflammatory response.^10^ Normally, pyrin is regulated by a RhoA-dependent phosphorylation and subsequent interaction with the 14-3-3 protein. An increasing body of evidence suggests that *MEFV* pathogenic variants lead to changes in pyrin that block phosphorylation sites for kinases, resulting in a lowered threshold for activation of the pyrin inflammasome.^11^ This leads to an increased secretion of IL-1b and IL-18, with the resulting pro-inflammatory effects.^12^

The disease may initially present in children with recurrent attacks of fever alone but typically progresses to more classic features such as pleuritis, peritonitis, and arthritis within 3 years of onset.^13^ Within the last decade, the potential clinical presentation has expanded and symptoms such as severe myalgia, protracted febrile myalgia syndrome, scrotal swelling, and cardiac involvement have been described in paediatric populations.^14^ According to new diagnostic criteria, diagnosis requires the presence of more than two of five major criteria (fever, abdominal pain, chest pain, arthritis, and family history of FMF).^15^ This new criterion has a higher sensitivity and specificity (86.5% and 93.6% in a Turkish population) than the previously used Tel Hashomer criteria.^16^ However, the figures have not been replicated in populations with lower incidences of FMF.

With respect to ocular involvement in FMF, the first observation was made in 1959, when Michaelson et al. noted dotted lesions on fundoscopy in Bruch’s membrane identified as colloid bodies.^17^ Since then, FMF has been linked to other ocular conditions such as acute posterior multifocal placoid pigment epitheliopathy and keratoconus where it may be a predisposing factor, especially if a patient is a carrier of a homozygous pathogenic variant.^18^ The most common ocular finding in a meta-analysis was the gradual decline in choroidal thickness, documented in 101 patients (47.9%).^19^ Retinal vasculitis was noted in 66 patients (31.2%), with rates of anterior uveitis of approximately 10%. A Turkish study in children with FMF found that during acute attacks, choroidal thickness was markedly increased compared to control groups as opposed to attack-free periods when there was no significant difference in thickness. This increase in choroidal thickness was also correlated with increased levels of inflammatory biomarkers, particularly C-reactive protein (CRP). On the one hand, the increased thickness was explained by the increase in inflammatory response and vascular permeability during acute attacks.^20^ Another possibility is that it is related to other changes such as increased body temperature during acute attacks. Nevertheless, a study in 61 patients with anterior uveitis concluded an association with increased subfoveal choroidal thickness.^21^ Of note, in a cohort of 32 patients with FMF, there was no difference detected in complement levels in FMF patients and healthy controls.^22^

## Tumour necrosis factor receptor-associated periodic fever syndrome (TRAPS)

TRAPS is an autosomal dominant MAIS linked to heterozygous variants of the *TNFRSF1A* gene responsible for generation of the Tumour Necrosis Factor Receptor 1 (TNFR1).^23^ TRAPS is the second most common MAIS, with an estimated prevalence of one per million, reported to occur more frequently in Caucasians.^24^ However, this may be due to ascertainment bias and unrepresentative of a true strong ethnic predominance as is true with FMF.^25^

In TRAPS, pathogenic variants in *TNFRSF1A* alter the extracellular domain of TNFR1, impacting structure and interaction with its ligand, TNF.^26^ Several molecular mechanisms have been proposed to explain the cellular disruptions involved in TRAPS, ultimately leading to pro-inflammatory cytokine production and recurrent fever. The pathogenic receptor can fail to undergo normal shedding from the cell surface, leading to a lack of soluble TNFR1 proteins that help dampen TNFR1 signalling.^27^ A further potential mechanism is the accumulation of misfolded proteins within cells, which induces endoplasmic reticulum stress leading to an unfolded protein response, and the elevated production of reactive oxygen species in mitochondria inducing pro-inflammatory cytokine production.^23^ Intracellular TNFR1 is also typically cleared through autophagy, a process that is also impaired in patients with TRAP further extending the activity of TNFR1.

Although clinical presentations can vary among different TRAPS phenotypes, a study involving a diverse group of 158 patients found that the median age for symptom onset was 4.3 years. Additionally, 9.1% of the patients experienced symptom onset after the age of 30.^25^ The most common clinical features included fever, limb pain, abdominal pain, rash, and ocular involvement. Symptoms such as lymphadenopathy, periorbital oedema, and abdominal pain were more likely in children. One of the most serious complications of TRAPS is AA amyloidosis and occurred in 10% of patients at a median age of 43 years in the above study.^24^ Over the past two decades, a series of diagnostic criteria have been developed to aid in the identification of TRAPS. These criteria focused on specific features including recurrent inflammatory symptoms, fever, abdominal symptoms, skin rashes, and lymphadenopathy in paediatric cases. In 2015, Gattorno et al. introduced an evidence-based classification system that aimed to integrate both genetic criteria (*TNFRSF1A* genotype) and specific clinical features to establish a diagnosis^1^ with increased sensitivity and specificity. The Eurofever panel (https://www.printo.it/eurofever/registry) recommended that for cases where genetic testing is unavailable, an alternative criterion based on clinical variables, with ordinal scores for fever, migratory rash, periorbital oedema, myalgia, positive family history, absence of aphthous stomatitis, and absence of pharyngotonsillitis should be used.

A systematic review across the MAIS literature found that across 17 studies, 138 patients with TRAPS experienced ocular involvement.^19^ The most common ocular manifestations were conjunctivitis (56.5%) and periorbital oedema (47.8%). Of note, two patients developed multifocal choroiditis (1.4%) and one patient developed bilateral panuveitis (0.7%). The case of panuveitis described a 7-year-old boy who presented with active bilateral panuveitis, with two choroidal lesions on the posterior pole of the right eye, and a macular rash associated with fever. They were subsequently diagnosed with TRAPS after genetic analysis found a pathogenic variant in the TNFRSF1A gene and treated with oral steroids and systemic immunosuppression.^28^

Interestingly, the patient was healthy until 6 months prior to the current evaluation. At that time, he developed bilateral acute conjunctivitis, fever, and a widespread maculopapular rash.

## Mevalonate kinase deficiency (MKD)

MKD is a recessively inherited pleiomorphic condition encompassing a range of diseases, (hyperimmunoglobulinaemia D [HIDS], periodic fever syndrome, and MVA [mevalonic aciduria]), with mild to severe complications.^29^ This group of phenotypes is caused by pathogenic variants in the mevalonate kinase encoding gene with varying degrees of severity according to varying levels of normal enzyme activity. MKD is one of the rarest MAISs with around 300 patients reported worldwide including 30 patients with MVA. Across the literature, a higher concentration of patients within European areas have been reported particularly in Western Europe and the Netherlands, where there is a particular incidence of HIDS.^30^ In comparison to 80 cases reported in The Netherlands, only 20 HIDS cases have been reported in the US. Whilst the data is sparse and studies are limited, an estimate for the prevalence of MKD has been estimated as 1.3 per million in European countries for patients younger than 19 years of age.^31^

Loss-of-function variants in the mevalonate kinase gene, which is responsible for generating the mevalonate kinase enzyme, have been linked to the aetiology of MKD. The mevalonate kinase enzyme follows HMG-CoA reductase (the target for statins) in the mevalonate pathway and converts mevalonic acid to 5-phosphomevalonic acid. The mevalonate pathway produces cholesterol while also generating nonsterol isoprene compounds. The reduced function of the enzyme leads to mevalonic acid accumulation and deficiency of downstream compounds. While the precise pathogenesis of MKD remains unclear due to a lack of representative models, several studies have convincing evidence suggesting that the pro-inflammatory state is secondary to reduced isoprene compounds.^29^ The use of statins to block the mevalonate pathway demonstrated that isoprenoid deficiency contributed to inflammasome activation and cytokine production. Additionally, approaches to increase cellular isoprenoid reduce inflammation in an experimental model.

The HIDS phenotype of MKD has been characterised as a milder manifestation presenting before 1 year of age, with recurrent bouts of fever, adenopathy, rash, and abdominal and joint pain, the latter potentially secondary to cholestasis and arthritis. The disease episodes are potentially cyclical and triggered by stress or vaccinations.^29^ A more severe form of MKD is MVA, also emerging in infancy with features such as frontal bossing, developmental delay, myopathies, and central nervous system involvement (e.g. psychomotor issues, ataxia, and seizures). HIDS and MVA are two extremes of a continuous spectrum of MKD disease. A diagnosis of MKD is typically confirmed by observing elevated IgD levels during flare-ups, reduced mevalonate kinase activity during symptom-free periods, and the presence of mevalonate in the urine. The Eurofever/PRINTO clinical classification criteria defined the following features to establish a diagnosis at least three of six criteria: age at onset <1 year, gastrointestinal symptoms, painful lymph nodes, aphthous stomatitis, triggers, and maculopapular rash.^5^ A recent study into the sensitivity and specificity of Eurofever MKD diagnostic criteria in a cohort of 119 patients found that the new criteria had a sensitivity and specificity of 87.5% and 60%.^32^ However, when this included genetic and clinical variables, it had a higher sensitivity and specificity of 85% and 100%, respectively.

The classical description of ophthalmological findings of MVA, the severe form of MKV, includes blue sclerae, uveitis, central cataracts, optic atrophy, and retinitis pigmentosa. In a meta-analysis of patients with MAISs, which included 32 cases with MKD, it was found that uveitis was more common in MKD than in any other MAIS (90.6%).^19^ Anterior uveitis (71.9%) was more common than intermediate uveitis (21.9%). A single case of early-onset bilateral granulomatous panuveitis with subsequent development of secondary glaucoma and total cataracts has been reported.^33^

## Cryopyrin-associated periodic syndrome (CAPS)

CAPS is a MAIS encompassing a range of diverse, autosomal dominant phenotypes caused by IL1ß-mediated systemic inflammation. The group includes familial cold autoinflammatory syndrome type 1 (FCAS1), Muckle-Wells syndrome (MWS), and neonatal-onset multisystem inflammatory disorder (NOMID). These conditions exist on a spectrum of severity with FCAS1 the least severe and NOMID the most aggressive.^34^ In contrast to FMF, CAPS does not exhibit significant ethnic predominance and while FCAS and MWS may be associated with familial inheritance patterns, NOMID is caused by random pathogenic variants.^35^

The uniting cause of all CAPS phenotypes is a pathogenic gain-of-function variant affecting the *NLRP3* gene.^36^ *NLRP3* codes for the NLRP3 (also called cryopyrin) protein, which normally functions as an intracellular pattern recognition receptor, are able to recognise PAMPS and DAMPS. The ensuing activated NLRP3 inflammasome comprises NLRP3, the adaptor protein ASC, and pro-caspase-1, which in turn activates the inflammatory cytokine, IL-1β. The pathogenic gain-of-function variant results in a loss of an autoinhibitory step in NLRP3 activation causing excessive generation of the active inflammasome without the presence of PAMPS and DAMPS stimuli.^37^

Due to the rare occurrence and diverse clinical presentation, diagnosing CAPS is challenging, leading to a significant delay between the onset of symptoms and the establishment of a definitive diagnosis.^36^ Symptoms of CAPS may present in acute attacks or because of organ damage due to chronic inflammation. Acute attacks are often triggered by a range of external factors including cold, stress, infections, trauma, or sleep deprivation. The clinical presentation of CAPS was first noted when reports of MWS in 1962 described the triad of urticaria, deafness, and amyloidosis.^38^ Subsequently, initial reports of NOMID in 1975 described a Still’s disease-like rash, deforming arthropathy, intellectual disability, and uveitis. A common symptom in all CAPS phenotypes is neutrophilic, non-symmetrical urticaria lasting 24 h. The rash is often non-painful but is burning and sensitive to touch. Whilst MWS generally presents in early childhood with a rash, arthralgia, myalgia, and fever during attacks, NOMID is more likely in early infancy with neurological symptoms common including chronic meningitis, increased intracranial pressure, developmental delay, seizures, and sensorineural hearing loss. The new Eurofever/PRINTO classification criteria in 2019 developed require the presence of a confirmatory NLRP3 genotype and at least one of the major features, namely: urticarial rash, red eye (conjunctivitis, episcleritis, uveitis), or sensorineural hearing loss. In the absence of the relevant NLRP3 genotype at least two of the features are required.^5^

A range of ocular manifestations have been described in CAPS. A study into a pool of 1353 patients with MAISs with ocular involvement found that conjunctivitis and papillitis were found significantly more often in CAPS compared to other MAISs.^19^ In this meta-analysis, 680 patients with CAPS were identified. The average age of onset for ocular involvement was 12.5 years, compared to a mean onset age of 5.5 years for CAPS itself. The most frequent ocular symptom was conjunctivitis (62.4%), followed by uveitis (28.4%). The breakdown of uveitis cases included 130 with anterior uveitis (19.1%), five with posterior uveitis (1%), two with intermediate uveitis and panuveitis (0.3%), and one with bilateral papillitis and uveitis (0.2%).

## Blau syndrome (BS)

Blau-Jabs syndrome, commonly referred to as Blau Syndrome (BS), is a rare autosomal dominant MAIS. BS is caused by pathogenic variants in the pattern recognition receptor Nucleotide-Binding Oligomerisation Domain-Containing Protein 2 (NOD2). The syndrome is characterised by the triad of arthritis, uveitis, and skin rash.^39^ BS/Early Onset Sarcoidosis (EOS) and systemic sarcoidosis are chronic granulomatous conditions displaying the common histologic feature of noncaseating granulomas, which can impact similar organ systems. Nonetheless, patients exhibit variations with respect to age of onset, genetic factors, and prevailing clinical characteristics. The incidence of BS remains uncertain. The yearly incidence of combined granulomatous disorders (encompassing BS, Early Onset Sarcoidosis, and Sarcoidosis) before the age of 18 has been documented at a range of 0.06 to 1.02 cases per 100,000 individuals.^40^ Individuals with BS typically manifest symptoms before reaching the age of 5, in contrast to children with “adult-type” sarcoidosis, who present during adolescence. BS predominantly affects children of Asian, Caucasian, or Hispanic heritage, while “adult-type” sarcoidosis emerging during childhood more commonly affects individuals of African American descent (80%).^41^ BS follows an autosomal dominant inheritance pattern, while EOS arises from a pathogenic variant that occurs sporadically in the same gene. The prevailing consensus indicates that the underlying pathogenesis is a NOD2 gain-of-function variant, resulting in the activation of proteins that would remain inactive under normal circumstances. NOD2 pathogenic variants cause increased generation of NOD2 protein, NF-κB activation, and proinflammatory cytokine production.

The clinical picture for BS is typified by a triad of symmetric arthritis, granulomatous dermatitis, and recurrent uveitis with onset below 4 years of age.^42^ A rash is often the first sign with papulonodular rashes and subcutaneous nodules being common. The arthritis resembles rheumatoid arthritis and can cause finger deformations and wrist stiffness. When making a diagnosis of BS, a choice of diagnostic procedures can be used to identify BS features. These include skin biopsy to identify noncaseating granulomas, and X-rays to identify carpal dysplasia, camptodactyly, abnormal ulna, and second metacarpal bone shape. Definitive confirmation of the disease is achieved through the genetic identification of pathogenic variants in NOD2.

In a meta-analysis of 238 patients with BS, the proportion of cases with uveitis was the highest of any other MAISs (95.4%).^19^ Of these cases 109 presented with granulomatous anterior uveitis (48%), 6 with intermediate uveitis (2.6%), 27 with posterior uveitis (11.9%), and 99 with panuveitis (43.6%). Ocular symptoms typically appeared in both eyes and often followed a chronic course of inflammation. These symptoms usually manifested later than joint and skin issues. Uveitis was consistently accompanied by either joint or skin symptoms, with no cases of isolated ocular disease reported.

Unfortunately, uveitis tends to be severe when present, with up to 30% of patients with BS developing moderate-to-severe visual impairment.^43^ In contrast to juvenile idiopathic arthritis (JIA)-related uveitis, which is more commonly non-granulomatous, BS typically presents as a bilateral panuveitis accompanied by peripheral multifocal choroidal scars.^43–45^

## Retinal dystrophy, optic nerve oedema, splenomegaly, anhidrosis, and headache (ROSAH)

Williams et al. identified a pathogenic variant, in the Alpha-protein kinase 1 (ALPK1) gene in 2019, associated with a condition marked by a range of symptoms including retinal dystrophy, optic nerve oedema, splenomegaly, anhidrosis, and headaches, which they termed ROSAH syndrome.^46^ The activation of the kinase domain in ALPK1 leads to the phosphorylation of the TRAF-interacting protein with a forkhead-associated domain, known as TIFA. This phosphorylation sets off a cascade that results in the formation of a TIFA–TRAF6 complex, ultimately leading to the activation of NF-κB signalling pathways. This process is crucial for innate immunity responses.^47^ The pathogenic variants identified in ROSAH appear to have gain-of-function with increased innate immune activation and enhanced NF-κB signalling.^48^

ROSAH is a newly described autosomal dominant MAIS, with few reports on identifying features and successful treatments. In the largest cohort of 27 patients, nearly all patients exhibited at least one inflammatory feature that included recurrent fever or abdominal pain, malaise, and headaches. The fever episodes were reported to predominantly last 24 h and self-resolve. Arthralgia was noted in 76% of participants, with cases of deforming erosive arthritis in 33%.

Ocular features included bilateral optic nerve swelling, which was nearly always present, and associated with uveitis during acute episodes, as well as progressive visual field loss. The mean age of presenting with visual symptoms was 15 years of age in this cohort.^49^ At early stages, the retina can appear normal; however, with more advanced disease, there has been evidence of progressive nummular retinal pigmentation, vascular attenuation, and retinal pigment epithelium atrophy.

## A20 haploinsufficiency (HA20)

A20 haploinsufficiency (HA20) is a rare autosomal dominant autoinflammatory disease caused by a heterozygous loss-of-function pathogenic variant in TNFα-Induced Protein 3 (*TNFAIP3*), which encodes for the NF-κB regulatory protein A20 or TNAP3.^50^ TNFAIP3 has a role in deubiquitinase activity and inhibits pro-inflammatory mediators such as NF-κB kinase subunit gamma and receptor-interacting protein kinase 1.^51^ HA20 was first described in six unrelated families with systemic inflammation in childhood, manifesting with symptoms similar to Behçet’s syndrome, with pathogenic variants causing defective deubiquitinase activity of A20, increased NF-κB signalling and phosphorylation of the c-Jun N-terminal kinase and p38 mitogen-activated protein kinases.^50^ In these families, five were described to have recurring Behçet’s disease and one patient from a Turkish cohort study carried pathogenic variants in *TNFAIP3*. Clinical features of Behçet’s syndrome and HA20 can be differentiated,^50^ and this distinction is key to treatment decisions, as for instance, response to colchicine in HA20 is less effective than in Behçet’s syndrome.^52^

Due to the rare occurrence of HA20, both its annual incidence and the details of its clinical symptoms, disease severity, systemic complications, and treatment approaches remain unclear. Further cases of HA20 have been reported in the literature, particularly in Japanese families, since the first description. From a Japanese family with three cases spanning three generations, two demonstrated an identical novel TNFAIP3 pathogenic variant.^53^ One of the family members had arthralgia, proximal limb muscle pain, recurrent aphthous stomatitis, aphthous ulcer of the palpebral conjunctiva and haemorrhoids. Another family member had polyarthritis and anterior uveitis starting in infancy and ultimately passed away at the age of 65 due to gastrointestinal bleeding. Another Japanese family investigated for presumed Behçet’s disease, with six patients over four generations presenting with frequent oral ulcers, genital ulcers, and erythema nodosum-like lesions but no ocular lesions, similarly identified a common heterozygous missense pathogenic variant in A20/TNFAIP3, with all carrying a specific heterozygous C234Y variant in the ovarian tumour domain.^54^ There is one case report of a germline heterozygous variant in TNFAIP3 causing A20 haploinsufficiency in a 7-month-old Japanese boy, with an unusual presentation of autoimmune lymphoproliferative syndrome, a condition characterised by chronic lymphoproliferation and autoimmunity.^55^ Expanding the clinical spectra of heterozygous loss-of-function pathogenic variants in TNFAIP3 is a case report of a 14-year-old British boy, presenting at age 10 with insulin-dependent diabetes, cytopaenias, hepatitis, enteropathy, and interstitial lung disease, who was responsive to haematopoietic stem cell transplantation.^56^ Some case reports have also described patients presenting with a lupus-like phenotype,^57^ inflammatory bowel disease,^58^ and haemophagocytic lymphohistiocytosis.^59^

Although no unifying diagnostic criteria exist, efforts have been made to improve the description of HA20, such as in a case series of 16 patients derived from seven families, in whom a genetic diagnosis of HA20 was established.^50^ In this case series, the age of disease presentation was highly variable, ranging from the first week of life to 29 years of age. Authors described the frequency and severity of clinical phenotype as variable, with early onset recurrent oral, genital, and/or gastrointestinal ulcers described as characteristic features of HA20. Other clinical manifestations reported include musculoskeletal symptoms, gastrointestinal complaints, cutaneous lesions, episodic fever, and recurrent infections. Of the ocular findings in this case series, severe and treatment-refractory uveitis in two sisters and retinal vasculitis with chorioretinal scarring and macular fibrosis and anterior uveitis in another young girl were described. In between flares, acute phase reactants were often within normal limits in most cases; however, there were some instances of increased levels of CRP and ESR pre-treatment, and the presence of autoantibodies was variable. In a meta-analysis of 89 patients, the median age of onset was 6 years old, with the main manifestations being recurrent oral ulcers (70%), recurrent fever (42%), gastrointestinal ulcers (40%), skin lesions (38%), genital ulcers (36%), and musculoskeletal disorders (34%).^60^

## Classification

Currently, there exists no clear consensus regarding the categorisation of MAIs. The 2022 updated phenotypic classification provided by the International Union of Immunological Societies expert committee on Inborn Errors of Immunity (IEI) is tailored towards clinicians at the bedside, focusing on the clinical attributes and laboratory features of distinct IEI conditions, including a section on auto-inflammatory disorders.^61^ Alternative classification methods have focused on a taxonomy dictated by molecular pathways involved,^62^ or the dermatological features that aid in differentiating the various conditions.^63^

For patients with MAISs, a useful classification strategy relies on identifying the dysregulated primary cytokine. This approach holds potential therapeutic value by allowing targeted intervention (Table 2). For this review, we will focus on this strategy further to emphasise potential therapeutic targets and treatments. Determining the predominant cytokine or pathway involved in the disease could pave the way for the creation of focused therapeutic approaches for MAISs.^64^

**Table 1.** Monogenic autoinflammatory syndromes clinical features.

Condition	Gene (MOI)	Protein	Clinical Features	Ocular Features	Onset^§^	Episode Duration	Episode Frequency
FMF	MEFV (AR or AD)	Pyrin	Polyserositis, Abdominal pain, Arthritis, Amyloidosis, Erysipelas-like erythema.	Vasculitis, Anterior Uveitis	3	1–4 days	Highly variable
TRAPS	TNFRSF1A (AD)	TNF Receptor Type 1	Prolonged Fever, Serositis, Rash, Amyloidosis, Joint Inflammation.	Conjunctivitis, Periorbital oedema/pain, Multifocal choroiditis	4	1–4 weeks	2–6 episodes/year
MKD	MKD (AR)	Mevalonate Kinase	Adenopathy, Oral Aphthosis, Diarrhoea. Mevalonate Aciduria during Attacks, Leukocytosis with high IgD levels.	Anterior Uveitis, Intermediate Uveitis, Cataracts	0.5	3–7 days	1–2 episodes/month
CAPS	NLRP3 (AD)	Cryopyrin (NLRP3)	Urticarial Rash, Sensorineural Hearing Loss, Fever, Arthralgia, Amyloidosis.	Conjunctivitis, Episcleritis, Uveitis	0.2	Persistent	Persistent
BS	NOD2 (AD)	TNF	Granulomatous Synovitis, Camptodactyly, Rash, Cranial neuropathies.	Granulomatous Uveitis	3	Persistent	Persistent
ROSAH	ALPK1 (AD)	ALPK1	Headaches, Recurrent Fever, Arthralgia,	Optic Nerve Oedema, Retinal Dystrophy	14	Persistent	Persistent
HA20	TNFAIP3 (AD)	TNAP	Recurrent Fever, Ulcers, Bloody Diarrhoea, Polyarthritis	Anterior Uveitis, Retinal Vasculitis and Choroiditis with Necrotising Inflammation	7	2–7 days	1–2 episodes/month

The major clinical features are described, with a focus on uveitis. Metrics including median age of onset, episode frequency, and duration are presented. Inheritance demonstrates differential penetrance, particularly in FMF, TRAPS, and CAPS. Clinical presentation is highly variable, with varying episode duration and frequency; AD: Autosomal Dominant, ALPK1: Alpha Kinase 1, AR: Autosomal Recessive, BS: Blau Syndrome, FMF: Familial Mediterranean Fever, HA20: Haploinsufficiency A20, MEFV: Mediterranean Fever Gene, MKD: Mevalonate kinase deficiency, MOI: Mode of Inheritance, NLRP3: Nucleotide-binding Domain, Leucine-rich–containing Family, Pyrin Domain–containing-3, NOD2: Nucleotide-Binding Oligomerisation Domain-Containing Protein 2, ROSAH: Retinal dystrophy, Optic nerve oedema, Splenomegaly, Anhidrosis, and Headache Syndrome, TNFAIP3: Tumour Necrosis Factor Alpha-Induced Protein 3, TNF: Tumour Necrosis Factor, TNFRSF1A: Tumour Necrosis Factor Receptor Superfamily Member 1A, TRAPS: TNF Receptor-Associated Periodic Syndrome, §: median age of onset in years. *References*.^4,5^

**Table 2.** Affected pathways and suggested treatments. Biologics are increasingly used upfront alongside non-specific targets such as steroids.

Condition	Cytokine Pathway	Targeted Therapy
FMF	IL-1β	Colchicine Steroids (for colchicine-resistant FMF) **Biologic** (for colchicine-resistant FMF or protracted febrile myalgia) with IL-1 targeted therapy: anakinra (daily), rilonacept (weekly), or canakinumab (bi-monthly) Adjuncts: NSAIDs (symptomatic relief)
TRAPS	TNF, IL-1β	Steroids (short-term control, successful in achieving remission in 40%) alongside biologic (reduce long-term side-effects; amyloidosis) **Biologic** First-line: IL-1 targeted therapy: anakinra (daily), rilonacept (weekly), or canakinumab (bi-monthly) Second-line: TNF targeted therapy: etanercept (not infliximab or adalimumab, due to paradoxical reactions) Adjuncts: NSAIDs (symptomatic relief)
MKD	IL-1β	Steroids (short-term control, successful in achieving remission only in 10%) alongside biologic **Biologic** First-line: IL-1 targeted therapy: anakinra (daily), rilonacept (weekly), or canakinumab (bi-monthly) Second-line: TNF targeted therapy: etanercept Adjuncts: NSAIDs (symptomatic relief)
CAPS	IL-1β	Steroids (short-term control, not successful in achieving remission) alongside biologic **Biologic** IL-1 targeted therapy: anakinra (daily), rilonacept (weekly) or canakinumab (bi-monthly) Adjuncts: NSAIDs (symptomatic relief)
BS	TNF	Steroids (acutely and long-term control) to achieve minimum dose with escalating non-steroid immunotherapy **Biologic** First-line: Anti-TNF: etanercept (weekly), adalimumab (bi-weekly), or infliximab (bi-monthly) Second-line: Consider IL-1 if remaining uncontrolled. Adjuncts: NSAIDs (symptomatic relief)

BS: Blau Syndrome, FMF: Familial Mediterranean Fever, TRAPS: TNF Receptor-Associated Periodic Syndrome, MKD: Mevalonate kinase deficiency. References:^48,65–68^

## Treatment

The evidence base for the treatment of monogenic autoinflammatory uveitides is primarily based on expert consensus and existing treatment regimens used to treat monogenic autoinflammatory diseases. Recent years have seen advancements in our understanding of the genetic underpinnings and pathogenesis of these conditions. Specifically, the role of the inflammasome in causing dysregulated production of IL-1ß has been clarified, particularly in the most commonly discussed autoinflammatory diseases.

## Conventional therapies

### Colchicine

Colchicine is an alkaloid extracted from Lily family plants *Colchicum autumnale* and *Gloriosa superba*. The use of colchicine in the treatment of gout has been recognised for centuries, but its use has also expanded to other conditions including Behçet’s disease, pericarditis, and cutaneous vasculitides.^69^ Several clinical trials have demonstrated the effectiveness of colchicine in treating Familial Mediterranean Fever (FMF), including its ability to reduce amyloidosis. These findings have led the European Alliance of Associations for Rheumatology (EULAR) to recommend colchicine as a first-line therapy for both adult and paediatric FMF patients.^70^

Colchicine has several anti-inflammatory effects. Primarily it functions via inhibition of leukocyte chemotaxis by an interaction with tubulin, leading to microtubule dysfunction. By binding non-polymerised tubulin, colchicine causes movement inhibition of intracellular granules. Other effects of colchicine include effects on TNF signalling by both reducing production by macrophages and effects of TNF receptors.^69^ It also inhibits phospholipase A2 activity, phagocytosis, and the release of lysosomal enzymes. Colchicine has also shown an ability to suppress the activation of caspase 1 leading to the inability to convert pro-IL-1 to active IL-1.^71^

Colchicine has a narrow therapeutic range due to a half-life following oral ingestion of between 7 and 9 h. It is metabolised in the liver and excreted in the biliary, intestinal, and renal systems. Its use in pregnancy and nursing patients is considered relatively safe, if hepatic and renal function is intact.^72^

### NSAIDs

Non-steroidal anti-inflammatory drugs (NSAIDs) exert their therapeutic effects via inhibition of cyclooxygenases, altering arachidonic acid in prostaglandins, and via thromboxanes. They have been used as symptomatic treatment of the monogenic autoinflammatory diseases either in isolation or as an additional therapy to other drug regimens.^65^ Although the Eurofever registry has documented complete response in a minority of patients treated with NSAIDs alone, they do appear to provide symptomatic benefit in 70–80% of patients.

### Corticosteroids

Corticosteroid therapies, such as prednisolone, have pleiotropic effects in control of inflammation via suppression of multiple pro-inflammatory pathways, direct effects of leukocyte migration, and inhibition of fibroblast function.^73^ This leads to disease control both acutely (less pain and oedema) and long term (reduced cell fibrosis associated changes). They exert their effect via binding to a steroid response element intracellularly leading to activation of a transcription factor and consequent gene expression effects: up-regulated anti-inflammatory proteins, and suppression of the pro-inflammatory NF-B pathway.

In FMF that is not sufficiently controlled with colchicine alone, the use of glucocorticoids can help further reduce disease activity and this aligns with data from the Eurofever registry.^65^ Acute control of disease can be further enhanced with the use of intravenous methylprednisolone during attacks of fever, abdominal pain, or pleuritic pain.^74^

Case reports of ocular involvement in patients with FMF described positive response to the addition of acute steroid therapy to manage uveitis flare-ups.^7^ In instances of anterior uveitis, positive outcomes were observed with the use of steroid drops. Alternatively, for intermediate or posterior uveitis cases, systemic steroids such as oral prednisolone or intravenous methylprednisolone were found to be effective.

In patients with TRAPS, the use of oral prednisolone can help relieve attacks with a 91% effectiveness in controlling inflammatory attacks.^65^ However, these treatments do not appear to alter the development of amyloidosis in these patients, nor do they reduce the frequency of attacks. Therefore, their use should ideally be limited to managing acute flare-ups.

Patients with TRAPS should be considered for biologic therapy to help minimise the ongoing steroid dose. During flare-ups, patients with MKD also respond positively to acute steroid therapy. However, an even smaller proportion achieve complete remission solely through steroid treatment compared to patients with TRAPS (9% vs 41%). In patients with CAPS, steroid therapy is also used with benefit in 80% of patients to provide additional relief during attacks; however, their use does not resolve the underlying inflammation or the frequency of attacks. In patients with CAPS, steroids should be ideally avoided as primary maintenance therapy.^75^

Patients with BS are commonly treated for ocular inflammation including uveitis and other systemic involvement with steroid therapy. A multicenter case series of 50 patients with BS worldwide identified 75% of eyes of patients treated with topical steroid drops, with 26 of 38 patients with ocular involvement treated with systemic corticosteroids.^44^ However, 76% of patients included in the case series continued to experience persistent uveitis activity despite the use of steroid therapies, with or without alternative immunosuppressants, reflecting the difficulty in controlling the disease entirely in this patient population.

### Thalidomide

Thalidomide has been used to treat dermatological diseases such as lupus and mucosal ulcers, in addition to its more common usage in multiple myeloma.^76^ Its anti-inflammatory properties arise from a mixture of inhibition of TNF-, interferon- (IFN-) synthesis, leukocyte chemotaxis, and angiogenesis. There have been sporadic case reports of its use in the treatment of the autoinflammatory monogenic including case series of colchicine-resistant FMF (crFMF) and MKD with no proven benefit.^77^ Thalidomide has also reportedly been used with success in four patients with Blau syndrome.^78^

### Other conventional medications

There is evidence from case reports of the use of azathioprine, methotrexate, cyclosporine, leflunomide, mycophenolate mofetil, dapsone, thalidomide, sulfasalazine, statins, cimetidine, and antihistamines in patients suffering from the autoinflammatory diseases.^65^

## Targeting the molecular mechanism: Biologic therapies

With understanding of the disease pathogenesis of the monogenic autoinflammatory diseases and further evidence from new clinical trials, newer more targeted approaches have been developed.

### IL-1 blockade

**Figure 2.**
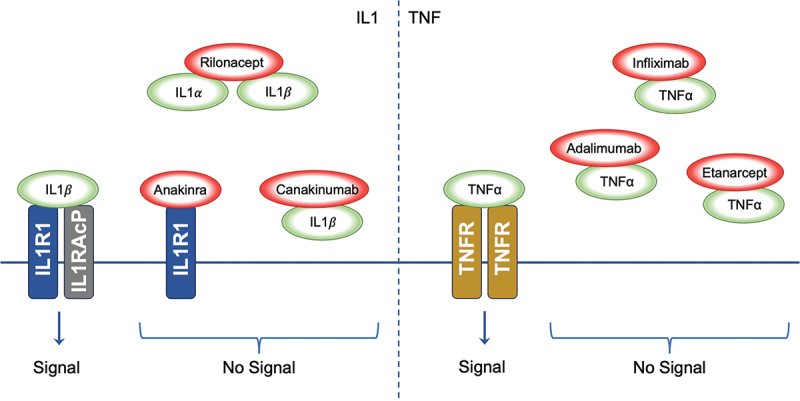
Inhibition of Cytokine Signalling by Therapeutic Agents. The left panel demonstrates the signalling cascade initiated by IL1 binding to the IL1R1-IL1RACP complex. IL1α and IL1β interaction with their receptor results in downstream signalling. The biologic agents Anakinra, Rilonacept, and Canakinumab disrupt IL1 signalling; Anakinra by antagonising receptor interaction, Rilonacept by sequestering IL1α and IL1β, and Canakinumab by neutralising IL1β, thus inhibiting the signal. The right panel shows TNFα signalling through the TNFR. The biologics Infliximab, Adalimumab, and Etanercept impede TNFα interaction with TNFR, thereby preventing signal transduction; IL1: Interleukin-1, IL1α: Interleukin-1 alpha, IL1β: Interleukin-1 beta, IL1R1: Interleukin-1 Receptor Type 1, IL1RACP: Interleukin-1 Receptor Accessory Protein, TNFα: Tumour Necrosis Factor-alpha, TNFR: Tumour Necrosis Factor Receptor.

The inflammatory cascade is triggered when the IL-1 receptor (IL-1 R) binds to the ligands IL-1α and IL-1β (Figure 2). IL-1 R is expressed on most human cells. IL-1 inhibitory molecules were first discovered in the urine of patients with monocytic leukaemia, and their use was hypothesised to be beneficial in treatment of patients with inflammatory conditions.^79^ This has been confirmed with substantial evidence supporting the use of IL-1 inhibitor therapy in inflammasome-driven diseases in particular.^65,79,80^ There are three commercially available IL-1 inhibitory therapies: anakinra, canakinumab, and rilonacept.

#### Anakinra

Anakinra is a competitive inhibitor of IL-1 R agonists which mimics the activity of endogenous IL-1 R antagonist (IL-1RA). It is a daily subcutaneous injection, with a half-life of approximately 6 h. It has received FDA approval for the treatment of NOMID since 2012, and EMA approval for all types of CAPS since 2013.^65^ It is 80% renally excreted hence requires preserved renal function when administered, and there is evidence from pre-clinical models it can cross the blood–brain barrier.^81^ Given long-standing usage in the treatment of patients with rheumatoid arthritis, there are good longitudinal safety data for its usage with no evidence of increase in both opportunistic infections and malignancies.^82^ Biologic registers do identify higher rates of serious skin infections and respiratory tract infections in patients prescribed anakinra, however.^83^ The most frequent adverse reaction is an injection site skin reaction, which tends to decline with time without the need for discontinuation. From limited registry data, there is no documented increase in congenital malformations or miscarriages when anakinra is administered in pregnant patients.

#### Canakinumab

Canakinumab is a fully humanised IgG1 monoclonal antibody specific for IL-1β.^80^ It is a bi-monthly subcutaneous injection, with an elimination half-life of 26 days. In 2016 based on the results of the CLUSTER trial, both the FDA and EMA approved the use of canakinumab in crFMF, TRAPS, and MKD.^84^ Canakinumab has a longer half-life than anakinra but does not appear to penetrate the blood–brain barrier. It is not influenced by renal function.^85^ There are limited data on the impact of canakinumab on pregnancy and breastfeeding. Adverse events relate to mild urinary and respiratory infections, although rarely serious infections have been reported in a CAPS registry; there is also a rare occurrence of neutropenia and thrombocytopenia in patients prescribed canakinumab.^86^

#### Rilonacept

Rilonacept is a dimeric fusion protein of the Fc portion of human IgG1 and of the human IL-1 receptor extracellular domain-binding IL-1/IL-1. It is a weekly subcutaneous injection, with a half-life of 7 days.^80^ It received FDA approval in 2008 for the treatment of FCAS/MWS in patients over the age of 12.^87^ As a large molecule is speculated not to cross the blood–brain barrier, it is likely excreted via the reticuloendothelial system as opposed to renally with dose adjustment not required in renal disease.^80^ Adverse events relate to local injection site reactions, headache, urinary and respiratory infections.

#### IL-1 blockade in autoinflammatory diseases

##### FMF

Anakinra is recommended for crFMF or experiencing protracted febrile myalgia. This is based on Eurofever registry data, and RCTs of anakinra demonstrating clear benefit.^70^ This was seen in patients prescribed canakinumab, and there is promising evidence of efficacy for rilonacept as well.

Although it may be possible to treat ocular manifestations of FMF through increasing Colchicine dosing alone,^88^ the use of anti-IL-1 therapies can help resolve resistant cases. Case reports also describe the use of IL-1 inhibitors to treat ocular complications of FMF; a case series described FMF-associated uveitis of unspecified subtype in two patients treated with canakinumab, one patient achieved remission whilst another had recurrence of uveitis after 10 months of therapy^89;^ a Turkish case report describes control of optic neuritis in a paediatric patient with crFMF with anakinra, which was then switched to canakinumab.^90^

##### TRAPS

IL-1 inhibitors have demonstrated efficacy in TRAPS and are superior to etanercept based on retrospective data.^65^ Anakinra has shown benefit in 90% of patients with TRAPS in registry data, and complete remission was observed in 67% of these and it is recommended for use in patients with TRAPS not controlled by NSAIDs or steroid therapy.^75^ Canakinumab has also shown significant efficacy with 19/20 patients exhibiting complete remission in an open-label trial,^91^ but similar to FMF patients on canakinumab, there were more adverse events than in placebo for these patients. The randomised placebo-controlled CLUSTER study trial further supported the use of canakinumab in the treatment of TRAPS with 45% of patients assigned treatment experiencing complete response compared to 8% in the placebo arm, which increased to 73% of patients on dose escalation in the trial.^84^ However, the specific treatment of ocular complications of TRAPS has not been described with IL-1 inhibitors.

##### MKD

IL-1 inhibitors can control or relieve symptoms in most patients. From registry data of 62 patients with MKD treated with anakinra, there was 84% response.^65^ A phase II study of canakinumab showed reduced frequency of attacks, normalised inflammatory markers, and complete clinical response in all patients. Based on the CLUSTER trial,^84^ FDA and EMA approval for canakinumab in MKD were granted with 35% achieving complete remission vs 6% on placebo, escalating to 57% of patients on an escalated dosing regimen. There is no specific reporting of the impact of IL-1 therapies on treating ocular manifestations of MKD; however, they are recommended as first line for both flares and long-term management of EULAR/ACR evidence-based expert consensus.^68^

##### CAPS

Anakinra, canakinumab, and rilonacept have all been approved by the FDA and EMA for CAPS and are typically first line therapy for all age groups of patients.^65^ For anakinra, a long-term open-label study of CINCA patients was the basis for approval by the FDA with symptoms and inflammatory markers improved.^92^ There was also evidence of improved leptomeningeal and cochlear involvement in these patients indicative of anakinra being able to cross the blood–brain barrier. Canakinumab was also effective in inducing remission in 75–90% of patients treated with it in the EuroFever registry, with similar levels of control to anakinra.^65^ An RCT of Rilonacept has also provided evidence of efficacy.^87^ While conjunctivitis is the more common ocular feature of CAPS, IL-1 inhibitors have also reported control of more severe ocular manifestations such as uveitis, with reported control of a severe granulomatous uveitis with stromal keratitis in a patient with CINCA after starting canakinumab^93^ and rapid control of a posterior uveitis in a patient with CINCA after starting anakinra.^94^ Four patients with MWS also had resolution of their uveitis after starting anakinra therapy in separate case reports.^95,96^

## Blau syndrome

Isolated case reports have reported rapid remission of uveitis in the context of treatment-resistant Blau syndrome with IL-1 inhibitors including anakinra^97^ and canakinumab.^98^

### TNF blockade

Anti-TNFs have been used to treat the monogenic inflammatory diseases, however there is poorer efficacy of these biologics compared to the IL-1 inhibitors.^65^ The agents used have included:

1. Etanercept – dimeric human TNF receptor p75-Fc fusion protein
2. Infliximab – chimeric monoclonal antibody against TNFα
3. Adalimumab – fully human mAb against TNFα

None of these therapies have received either FDA or EMA approval for their use in the monogenic autoinflammatory diseases.

#### Anti-TNFs in autoinflammatory diseases

##### FMF

There is evidence from case series reporting some benefit when used to treat CRFMF, especially when other autoimmune comorbidities are present, e.g. ankylosing spondylitis or psoriasis.^99,100^ This has also been identified in registry data with more benefit in patients with co-incident arthritis.^65^

##### TRAPS

Etanercept has reported some benefit in reducing severity of attacks and helping reduce ongoing steroid dosing in patients with TRAPS.^65^ However, it is often discontinued due to lack of effect over time, which is the basis for consensus recommendations for use in some TRAPS patients.^75^ The other anti-TNFs infliximab and adalimumab have been associated with severe paradoxical reactions and are not recommended for use in patients suffering from TRAPS.^101^ Anti-TNFs have efficacy in managing the systemic disease, which should guide management given that the main ocular manifestations of TRAPS are conjunctivitis or periorbital oedema. However, a single case report does describe a patient presenting with fever and bilateral panuveitis diagnosed with TRAPS with genetic testing who achieved sustained control of inflammation for 16 months follow-up whilst on adalimumab therapy.^28^ Similar to patients with JIA continuing to experience new or ongoing flares of uveitis on etanercept,^102^ etanercept has also been linked to the incidence of anterior uveitis in patients with TRAPS treated with it^103^; its use should therefore be re-evaluated to consider stopping or switching to an alternative biologic in patients with TRAPS that suffer uveitis whilst prescribed etanercept. Compared to patients treated with anakinra, TRAPS patients are less likely to evidence complete remission on anti-TNFs.

##### MKD

Anti-TNFs have been documented in case reports to improve symptoms and reduce the frequency of attacks of MKD.^75^ Anti-IL1 therapies remain first-line with greater control of symptoms reported in case series,^104^ however anti-TNFs can be considered in cases where anti-IL-1 therapies are ineffective.^68^ Etanercept is the most prescribed anti-TNF for this purpose based on registry data.^65^ The use of anti-TNFs to treat uveitis related to MKD has been described in a case report of a 2-month-old boy followed up for 7 years after presenting with bilateral panuveitis, which was managed with steroids, methotrexate, and adalimumab but continued to experience ongoing uveitis flare-ups.^33^ However, a patient with corneal inflammation secondary to MKD, with a recurrent nummular keratitis, experienced significant improvement of their symptoms and cessation of keratitis flare-ups following a regimen including infliximab and methotrexate therapy.^105^

##### CAPS

There is no documented evidence to support the efficacy of treatments for CAPS syndrome.

## Blau syndrome

Anti-TNF therapies have demonstrated efficacy from multiple case reports and case series of patients diagnosed with BS and have also been identified as effective in the control of uveitis secondary to BS. Based on a review of 38 published case reports of BS,^106^ 62 patients were treated with good disease control on anti-TNF therapy in 27 of 31 patients treated with infliximab, 21 of 24 patients treated with adalimumab, and 5 of 7 patients treated with etanercept.

### IL-6 blockade

Tocilizumab is a humanised anti-IL6 receptor antibody which has been approved for use in RA, JIA, GCA, and as a treatment for the CAR-T therapy associated cytokine release syndrome.^107^ There is currently limited experience of its use in the treatment of the MAISs; hence, there are no current approvals. However, IL-6 inhibition may provide additional benefits, especially in cases of monogenic autoinflammatory disease not well controlled with other therapies. The use of Tocilizumab has been reported in the treatment of crFMF with evidence of symptom relief and control of amyloidosis.^108^ It has also been used in case series of TRAPS and HDS/MKD patients with some evidence of improvement.^107,109^ There have been two reported negative results with patients treated with Tocilizumab for CAPS with initial control followed by rapid relapse in both.^110,111^

There is also a single-case report from China of a 13-year-old patient diagnosed with BS suffering treatment-resistant uveitis, which was managed with tocilizumab, helping taper their steroid therapy to a much lower ongoing dose,^112^ although wider usage of IL-6 inhibition in BS has not since been reported.

### JAK inhibitors

JAK kinase inhibitors suppress the STAT1 transcription factor pathway which blocks the induction of IFN stimulated genes and hence reduces the production of IFN. The JAK inhibitors include tofacitinib, baricitinib, and ruxolitinib and have been approved for the treatment of rheumatoid arthritis, psoriatic arthritis, IBD, myelofibrosis, and polycythaemia rubra vera.^113^ There are reports of use of the JAK inhibitor tofacitinib in the treatment of crFMF patients who failed IL-1 inhibitor, anti-TNF, and IL-6 inhibition.^114^ JAK inhibitors have been used to treat patients with BS with tofacitinib inducing remission in three patients unresponsive to initial therapies including anti-TNF therapy.^115^ Another case report of a patient with anti-TNF refractory BS described remission once prescribed tofacitinib, which was then switched to baricitinib for maintenance therapy due to tofacitinib-induced lymphopenia.^116^

## Future directions

As our understanding of the inflammasome grows, new avenues for disease treatment are emerging. Specifically, we can now target oxidative stress, autophagy, and the complement cascade to alter the course of the disease.^117^

There remains a need for higher quality evidence from clinical trials to inform management of patients with MAIs. To this end, the development of shared clinical databases to further characterise response in affected patients to newer biologic therapy regimens will continue to provide valuable real-world data of the impact of therapies for patients with these rare diseases.
